# 4114 A Community/Academic Partnership to Implement Nutritional and Social/Behavioral Interventions to reduce Hypertension among Seniors Aging in Place

**DOI:** 10.1017/cts.2020.248

**Published:** 2020-07-29

**Authors:** Kimberly Vasquez, Andrea Ronning, Moufdi Naji, Glenis George-Alexander, Clewert Sylvester, Cameron Coffran, Teeto Ezeonu, Chamanara Khalida, Jonathan N. Tobin, Dozene Guishard, Rhonda G Kost

**Affiliations:** 1 Rockefeller University; 2 Carter Burden Network; 3 Clinical Directors Network

## Abstract

OBJECTIVES/GOALS: The Rockefeller University CCTS, Clinical Directors Network (CDN), and Carter Burden Network (CBN) received a DHHS-Administration for Community Living Nutrition Innovation grant to test whether implementation of DASH-concordant meals and a program to enhance self-efficacy, could lower blood pressure among seniors aging in place. METHODS/STUDY POPULATION: CEnR-Nav model to engage stakeholders, enroll seniors age >60 yr., eating 4 meals a week at 2 CBN congregate meal sites; Advisory Committee to facilitate dissemination; menus aligned with Dietary Approaches to Stop Hypertension (DASH) and New York City Department for the Aging (DFTA) nutritional guidelines; interactive sessions for education (nutrition, blood pressure, medication adherence); Omron 10 home BP devices for daily home monitoring. Plate Waste and Meal Satisfaction (Likert scale) to assess taste preference and cost impact. Outcomes: *Primary:* Change in Systolic BP at Month 1; change in percent with controlled blood pressure. *Secondary:* change in validated measures of cognitive (e.g. SF-12, PHQ-2), behavioral (Home BP monitoring), nutritional (food frequency) variables, satisfaction, costs. RESULTS/ANTICIPATED RESULTS: Menu alignment required multiple iterations. Plate Waste and Menu Satisfaction tools were developed. Site 1 enrollment began June 2019; educational sessions and home BP monitors and training were provided. Baseline mean blood pressure (Site 1) was 138/79 +20.5; (range: 7% hypertensive crisis, 36% stage 2 hypertension, 22% stage 1 hypertension, 22% elevated, and 13% normal). DASH-aligned meals began October 2019; Meal satisfaction declined briefly, chefs adjusted menus, and meal satisfaction rose to pre-intervention levels. Site 2 enrollment is ongoing; dietary intervention will start in 2020. Primary outcome data (change in BP) will be complete in March 2020. Secondary outcome data on social and behavioral impact of the interventions will also be presented. DISCUSSION/SIGNIFICANCE OF IMPACT: We leveraged our community-academic research partnership to conduct research addressing uncontrolled hypertension, an urgent unmet health need among seniors. The DASH Implementation Study can inform the broader aging services and healthcare community of the potential for congregate nutrition programs to improve cardiovascular health outcomes.

